# The Effect of Muscle Exercise on Perforators Flow: A Prospective Cohort Study

**DOI:** 10.3390/medicina56070338

**Published:** 2020-07-08

**Authors:** Francesco Amendola, Luca Vaienti, Giuseppe Cottone, Giovanna Zaccaria, Zvi Steinberger, Nicole Dereatti, Michele Riccio, Glenda Giorgia Caputo, Francesco De Francesco, Nicola Zingaretti

**Affiliations:** 1 Plastic and Reconstructive Surgery Department, I.R.C.C.S. Policlinico San Donato, Piazza Edmondo Malan, 2, 20097 San Donato Milanese, Italy; fgsamendola@gmail.com (F.A.); luca.vaienti@unimi.it (L.V.); gcottone.md@gmail.com (G.C.); 2 Department of Plastic and Reconstructive Surgery, Policlinico di Modena Azienda Ospedaliero-Universitaria di Modena, Via Largo del Pozzo, 71, 41125 Modena, Italy; zaccariagiovanna1982@gmail.com; 3 Hand and Microsurgery Department, Derech Sheba 2, 52621 Ramat Gan, Israel; Steinberger.zvi@gmail.com; 4 School of Medicine, University of Udine, 33100 Udine, Italy; nicole.dereatti@gmail.com; 5 Department of Reconstructive Surgery and Hand Surgery, AOU “Ospedali Riuniti”, 60126 Ancona, Italy; michelericcio.dr@gmail.com (M.R.); fran.defr@libero.it (F.D.F.); 6 Clinic of Plastic and Reconstructive Surgery, Department of Medicine (DAME), University of Udine, Piazzale Santa Maria della Misericordia 15, 33100 Udine, Italy; glendagiorgia.caputo@asufc.sanita.fvg.it

**Keywords:** perforator, flow, muscle exercise, anterior tibial artery perforators, hemodynamic flow

## Abstract

*Background and objectives*: The metabolic response after exercise causes a significant increase in the muscle blood flow. While these effects are demonstrated for intra-muscular vessels, there is no evidence about the inter-muscular vessels, such as the septocutaneous perforators supplying the skin after they branch out from the deep source artery. The aim of our prospective study was to quantify the changes in the anterior tibial artery perforators arterial blood flow after mild isotonic exercise in a young and healthy population. *Material and Methods*: We performed a prospective analysis of 34 patients who were admitted to the Plastic Surgery Department from December 2019 to April 2020. Flow velocities of two previously identified anterior tibial artery perforators were recorded both before and after 10 complete flexion-extensions of the foot. The time to revert to basal flow was measured. We further classified the overmentioned patients based on their level of physical activity. *Results*: We registered a significant increase in systolic, diastolic and mean blood flow velocities both in proximal and distal anterior tibial artery perforators after exercise. Fitter patients exhibited a higher increase in proximal leg perforators than those who did less than three aerobic workouts a week. The time to return to basal flow ranged from 60 to 90 s. *Conclusions*: This was the first study to describe the effect of muscular activity on perforators blood flow. Even mild exercise significantly increases the perforator flow. Waiting at least two minutes at rest before performing the Doppler study, thus avoiding involved muscle activation, can notably improve the reliability of the pre-operative planning.

## 1. Introduction

Extensive soft tissue defects of the lower leg, especially in the distal third, are challenging for surgeons [1,2,3]. Local perforators or distant free flaps are often required to achieve full reconstruction [4].

The introduction of perforator flaps constituted a significant step forward in the reconstructive microsurgery field. They are easy to handle, as well as safe, even in cases of challenging and complex reconstructions. They also have several advantages compared to traditional flaps: a better control of the flap thickness with the ability to harvest significantly thinner flaps and the disappearance of the “flap of choice” concept, since the great versatility of these flaps makes the entire body a potential donor site.

According to the definition of the Gent International Course on Perforator Flaps [5], a perforator flap is composed of skin and subcutaneous tissue, nourished by perforator vessels arising from the deep vascular system.

Running through muscles or between intermuscular septa, a perforator pierces the fascia to provide a specific blood supply [6] to the overlying skin. Perforator flaps in the lower leg mainly originate from tibial and peroneal arteries.

The anterior tibial artery (ATA) arises from the popliteal artery, just distal to the popliteal muscle. It passes between the two heads of the tibialis posterior and enters the extensor compartment, crossing the interosseous membrane. Along its course, the ATA gives out 3–4 perforators on average, both musculocutaneous and septocutaneous [7,8]. Morrison and Shen [9] demonstrated that in most cases the main perforator is a septocutaneous vessel that originates within 7 cm from the fibular head and penetrates the deep fascia through the anterior intermuscular septum between the extensor digitorum longus and peroneus muscles.

Doppler ultrasonography is the cornerstone of perforator flap surgery; perforators are identified during the pre-operative planning guide flap design, as well as during the surgical approach. Having great accuracy during pre-operative planning saves time, eases flap elevation and may help in avoiding unnecessary exploration [8]. We place great emphasis on getting to know perforators blood flow variations to ensure the best surgical outcome with no complications.

Physiologically, the arterial blood flow through a muscle is influenced by local metabolic factors [10,11,12,13,14]. During exercise, muscle metabolic activity can increase more than 60-fold and the blood flow can increase as much as 20-fold. On the other hand, skin blood flow is controlled largely by the central nervous system through the sympathetic nerves [15]. Hence, we do not expect any significant increase in cutaneous blood flow during mild local muscular exercise.

Having established that skin is nourished by perforator vessels running between or within muscles, we wondered how the skin perforators blood flow would change in response to the activation of those same muscles.

No study has been performed to analyze the effects of muscle activation on the local perforator vessels and how these changes can affect the surgical approach.

The aim of this study was to analyze blood flow changes registered in ATA perforators after isotonic contractions of extensor muscles of the foot. In addition, we aim to quantify the blood flow change and to measure the average time to revert to basal conditions. An acutely increased blood flow could alter the reliability of the pre-operative Doppler analysis in perforator flap-based reconstruction. Performing the ultrasound analysis after the “recovery time” might increase the accuracy of the exam.

## 2. Materials and Methods

In accordance with our study protocol, the Helsinki Declaration of 1975 and under Local Ethical Committee approval (IRB—EGAS CEUR: CEUR-2020-Sper-054), we prospectively collected and analyzed data from 34 young healthy patients referred to our medical institution for a first consultation in plastic surgery. All patients enrolled voluntarily and without any financial compensation. Written informed consent was obtained from all patients in the study.

The data were collected from January 2020 to May 2020.

The main outcome was to demonstrate an increase in ATA perforators blood flow after 10 active flexion-extensions of the foot. Secondary outcomes were the possible differences in blood flow increase between the proximal and the distal perforators and the average time needed to return to the basal flow.

We proposed enrollment in the study to all our patients who were referring for minor outpatient cutaneous surgery. Every patient meeting the inclusion criteria was consecutively enrolled in the study.

Patients were enrolled with the following inclusion criteria: aged 18–40 years; BMI > 18.5 and <25 kg/m^2^.The exclusion criteria were: positive history for lower leg trauma; prior surgical intervention on the lower limb; patients diagnosed with diabetes, arterial hypertension or any type of systemic comorbidities; consumption of any type of medication or recreational drug; arterial or venous vasculopathies; female patients during the last 7 days of the predicted end of their period.

After a preliminary statistical power analysis, setting the effect size to 0.5, beta to 0.2 and alpha to 0.05, we calculated a sample size of 34 patients.

Detailed social, pharmacological and medical history was taken from each patient. Attention was posed to smoking habits and physical activity. The latter was categorized as absent, low (less than two aerobic trainings of at least 30 min during the week) and high (more than two aerobic trainings per week).

### 2.1. Clinical Examination

The perforator arterial flow was assessed with handheld Doppler ultrasonography (8 mHz, Basic 3.1, Atys Medical, Soucieu en Jarrest, France). Systolic, diastolic and mean arterial flow velocities in meters per second were recorded. For every patient, only the dominant lower extremity was included in the study. The length of the leg was measured from the fibular head to the lateral malleolus. We included two perforators for each leg, assuming that the proximal one had a higher probability to be musculocutaneous, and the distal to be fasciocutaneous.

**Figure 1:**
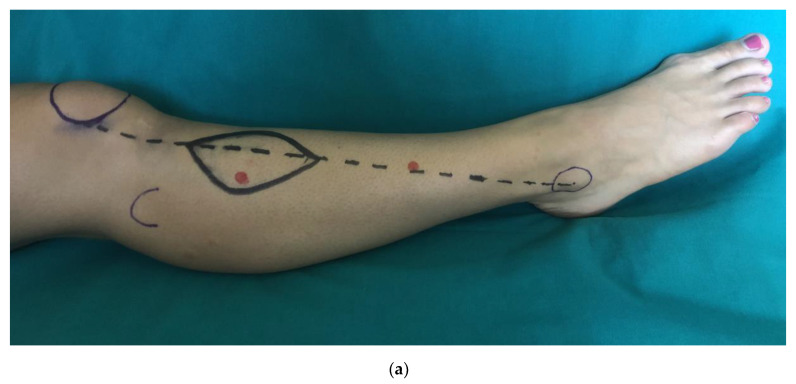

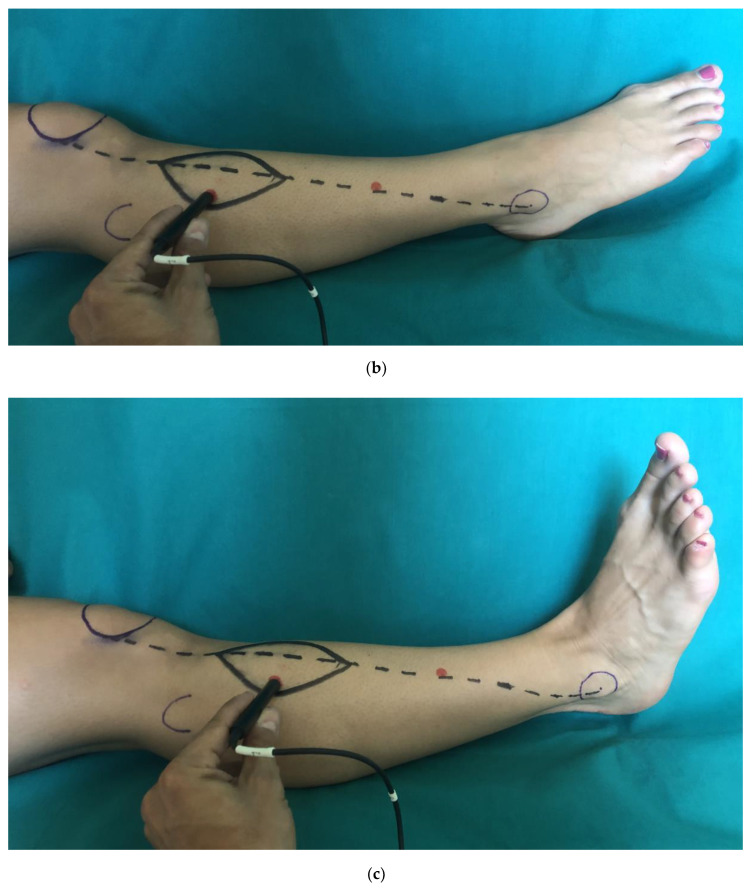

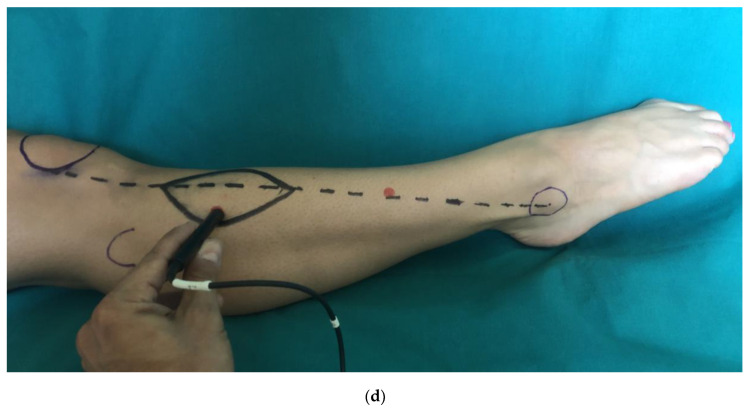
Data collecting protocol to find differences in the anterior tibial artery (ATA) perforators blood flow after 10 active flexion-extensions of the foot. Identifying two ATA perforators on the dominant leg with US (**a**), (one in the proximal half and one in the distal half, in red points) of a supine resting patient and marking them on the skin. Construction of ATAP flap by defining a meridian from the midlateral aspect of the patella to the midpoint of the lateral malleolus (dotted black line). Recording the flow variables at rest for the proximal perforator (**b**). Instructing the patient to perform ten complete dorsi-plantar flexions (**c**,**d**); then recording the flow variables after the exercise and the time in seconds needed to return to basal conditions.

The data collecting protocol was as follows: (1) identifying two ATA perforators on the dominant leg (one in the proximal half and one in the distal half) of a supine-resting patient and marking them on the skin (Figure 1a); (2) recording the flow variables at rest for the proximal perforator (Figure 1b); (3) instructing the patient to perform ten complete dorsi-plantar flexions (Figure 1c,d); (4) recording the flow variables after the exercise (5); recording the time in seconds needed to return to basal conditions. The same procedure from point 2 was repeated for the distal perforator.

We choose the flexion-extensions of the foot to reduce confounding bias. As previously described for the passive limb movements and their effect on the peripheral vascular response [16,17], mild local muscle exercises do not significantly affect the cardiac output. With stable conditions in heart rate and blood pressure, we improved the selectivity of our analysis and focused on the local metabolic factors of muscles surrounding the perforators.

### 2.2. Statistical Analysis

Distribution of continuous data was tested with the Shapiro–Wilk test. Normally distributed variables were expressed as mean ± standard deviation, whereas non-normally distributed variables were presented as median and interquartile range. Continuous variables were then compared using paired-sample Student’s t test or Wilcoxon signed-rank test.

## 3. Results

Twenty males and fourteen females were included in the study (Table 1). The mean age was 27 years (±5 SD). Eight patients were active smokers. Eight patients admitted to not have physical activity, 18 were classified as having a low intensity physical activity and 8 as having a high intensity physical activity.

The proximal anterior tibial artery perforator was found on average 4 cm distally to the head of the fibula, while the distal one was on average 9 cm proximally to the lateral malleolus.

At rest, the median values of systolic, diastolic and mean flow velocities were 16 m/s (IQR 13–20), 2 m/s (IQR 1–2) and 4 m/s (IQR 2–4), respectively for the proximal perforators; 16 m/s (IQR 13–20), 1 m/s (IQR 1–1) and 2 m/s (IQR 2–3), respectively for the distal perforators.

After exercise, the median values of systolic, diastolic and mean flow velocities were 33 m/s (IQR 28–42), 16 m/s (IQR 14–19) and 17 m/s (IQR 12–20), respectively for the proximal perforators; 27 m/s (IQR 22–34), 9 m/s (IQR 7–13) and 10 m/s (IQR 7–12), respectively for the distal perforators. All of the differences between pre- and post-exercise values of flow were statistically significant with a *p*-value of <0.001.

We then further stratified the physical activity. The median increase in the mean blood flow velocity in proximal perforators was significantly higher for the highly active patients compared to the non-highly active patients (8-fold versus 5-fold increase, *p* = 0.02). The same comparison for the distal perforators was not significant (5-fold versus 4-fold increase). No significant differences were encountered between smokers and non-smokers.

The median time needed to return to basal flow was 70 s (IQR 60–80) for proximal perforators and 80 s (IQR 70–90) for distal perforators. Details about all of the measures are outlined in Table 2.

## 4. Discussion

Lower leg reconstruction often requires the use of local perforator flaps, nourished by either the tibialis arteries or the peroneal artery perforators. Propeller and advancement flaps are the most commonly used local flaps in lower extremity reconstruction and they often constitute the final reconstructive option before moving to a distant free flap. Doppler ultrasonography is a fundamental tool during perforator flap surgery planning [18]. Exact concordance between pre-operative and intra-operative surgical findings speeds up the reconstruction, facilitates the surgeon’s work and helps in avoiding unnecessary surgical exploration.

Song et al. [19] reported a different flow in perforators of propeller fasciocutaneous flaps depending on the arc of rotation. The higher flows registered adopting the correct rotation implied that flow variations might alter both the pre-operative planning and the surgical outcome.

Our results were consistent with the well-known vessel physiology, demonstrating a vasodilation and an increase in blood flow following exercise [12,20]. Nevertheless, the correlation between muscular exercise and the increase in skin perforator blood flow is described here for the first time.

In our work, we demonstrated a significant blood flow increase in ATA perforators after as few as ten foot flexion/extensions in a healthy and young population. A significant increase in systolic, diastolic and mean blood flow velocities was measured both in proximally and distally located perforators.

Interestingly, fitter patients performing more than three aerobic workouts a week showed a significantly higher increase in mean blood flow velocity in proximal perforators. Distal perforators did not show a similar increase. The overmentioned increase might be explained by a greater vascular response in more trained muscles. The absence of muscle bellies at the distal third of the lower leg might, on the other hand, explain the lack of differences in flow increase between more and less trained patients.

Eventually, we observed that the time needed to return to basal flow ranged from 60 s to 90 s. A few steps taken by patients before the pre-operative Doppler analysis (searching for perforator vessels) could be enough to increase the perforators flow, thus altering the planning itself. We therefore advise to perform Doppler ultrasonography at least 90 s after the patient is completely at rest.

The main limits of this study were the relatively small cohort, as well as the obviously different perforators anatomical locations in the leg between patients.

We advocate further clinical studies with larger cohorts that include direct measurements of vessel caliber changes with exercise. Moreover, we encourage additional studies looking for the appearance of new perforators after an exercise-induced flow increase, which were not detectable at rest. Eventually, the demonstrated increase in perforator blood flow should be clinically exploited to unmask a possible therapeutic role of muscle exercise in perforator flap arterial insufficiency.

## 5. Conclusions

This was the first study in literature correlating perforators blood flow to muscle exercise. Just ten flexion-extensions of the foot significantly increased the anterior tibial artery perforators flow. During the planning of perforator-based reconstructions, performing the pre-operative ultrasound analysis immediately after even a minor muscular exercise of the foot can increase false positive results, thus reducing the reliability of the pre-operative planning.

## Figures and Tables

**Table 1:** Patient demographics of the study population.

Patient	Sex	Age (y)	Physical Activity	Smoking
1	M	28	low	no
2	F	27	high	yes
3	M	35	low	no
4	M	30	high	no
5	M	27	high	no
6	F	31	low	no
7	M	25	none	no
8	M	25	none	no
9	F	25	none	no
10	M	26	high	no
11	M	27	high	no
12	M	27	low	no
13	M	23	low	no
14	M	24	low	no
15	M	23	low	no
16	M	23	low	yes
17	M	24	low	no
18	M	27	high	no
19	F	21	low	no
20	F	28	low	no
21	F	24	none	no
22	M	24	none	yes
23	F	25	low	no
24	F	38	high	yes
25	F	24	low	yes
26	F	24	none	no
27	M	37	low	yes
28	F	24	low	no
29	F	25	low	no
30	F	40	high	no
31	M	32	low	no
32	M	21	none	yes
33	F	37	low	no
34	M	24	none	yes

**Table 2:** Patients’ characteristics about flow variables pre- and post-exercise.

	Proximal Perforator						Distal Perforator					
	Systolic Velocity		Diastolic Velocity		Median Velocity		Systolic Velocity		Diastolic Velocity		Median Velocity	
Pt	Pre	Post	Pre	Post	Pre	Post	Pre	Post	Pre	Post	Pre	Post
1	15	18	1	14	2	17	18	30	1	11	3	12
2	12	33	1	18	2	17	13	28	1	16	1	19
3	14	70	2	30	3	30	20	25	1	12	1	12
4	14	50	1	25	2	30	20	44	1	15	2	20
5	20	50	1	28	3	23	12	18	5	16	3	12
6	15	32	1	22	3	19	12	17	1	9	1	7
7	23	31	9	16	10	15	30	34	6	5	6	4
8	11	19	1	12	1	12	30	36	1	11	5	10
9	16	56	1	17	2	26	15	23	1	7	2	9
10	13	29	1	10	1	12	25	40	1	0	2	5
11	8	36	3	18	1	14	23	17	1	3	2	1
12	13	38	3	28	3	20	22	23	3	13	2	12
13	22	35	1	15	3	14	19	19	1	2	2	3
14	17	52	1	16	3	18	21	28	1	4	2	8
15	19	42	1	16	3	18	16	16	1	7	2	7
16	21	26	1	7	4	10	14	23	1	8	1	4
17	13	19	1	7	2	7	16	65	1	10	2	21
18	12	29	1	17	3	18	16	30	1	14	2	15
19	23	42	8	54	8	33	11	41	1	21	2	19
20	33	44	6	34	8	32	17	21	1	9	3	7
21	20	36	7	19	8	5	10	20	1	9	3	7
22	28	34	1	4	6	9	18	42	3	8	4	9
23	12	25	2	13	4	8	16	23	1	10	2	11
24	16	29	1	18	3	16	11	22	1	9	2	10
25	17	27	1	14	3	16	19	39	1	16	3	19
26	16	18	1	6	4	8	11	22	1	6	2	10
27	11	28	1	14	1	11	16	24	3	9	3	10
28	18	33	1	7	3	8	16	18	1	9	2	7
29	12	41	1	14	2	18	21	32	1	5	2	7
30	15	47	2	17	3	19	17	29	1	11	2	12
31	17	25	1	16	5	24	14	34	1	12	2	11
32	16	31	1	15	5	23	13	39	2	13	3	8
33	16	28	2	15	2	13	13	23	1	9	3	12
34	21	38	1	20	3	16	14	31	1	13	3	11

Pt: patient; Pre: before exercise; Post: after exercise.
